# Peripartum fetal distress in diabetic women: a retrospective case-cohort study

**DOI:** 10.1186/s12884-018-1880-4

**Published:** 2018-06-14

**Authors:** B Castelijn, KWP Hollander, JF Hensbergen, RG IJzerman, AW Valkenburg-van den Berg, JWR Twisk, CJM De Groot, MGAJ Wouters

**Affiliations:** 1 Department of Obstetrics and Gynecology, VU University Medical Center, PO Box 7057, Amsterdam, 1007 MB the Netherlands; 2 Department of Obstetrics and Gynecology, Onze Lieve Vrouwe Gasthuis, Amsterdam, the Netherlands; 3 Department of Internal Medicine, VU University Medical Center, Amsterdam, the Netherlands; 4 Department of Epidemiology & Biostatistics and Department of Health Sciences, VU University, Amsterdam, the Netherlands

**Keywords:** Type 1 diabetes, Type 2 diabetes, Gestational diabetes, Fetal distress, Umbilical artery pH

## Abstract

**Background:**

Major concerns of pregnancies complicated by diabetes mellitus are an increased risk of adverse perinatal outcome. The objective of this study was to analyse the rate of fetal distress during labor in women with type 1, type 2 and gestational diabetes compared to control women.

**Methods:**

A retrospective case-cohort study was conducted at the VU University Medical Center, Amsterdam; a tertiary care hospital. 117 women with type 1 diabetes, 59 women with type 2 diabetes, 303 women with gestational diabetes and 15,260 control women were included, who delivered between March 2004 and February 2014. Linear and logistic regression analyses were used to compare maternal and pregnancy characteristics. Risk of fetal distress and perinatal asphyxia was assessed by multiple regression analyses, adjusted for confounding factors as age, smoking, parity, previous cesarean section, hypertensive disorder, pre-eclampsia, prematurity, induction of labor and macrosomia. Main outcome measure was fetal distress, defined either as clinical indication for instrumental or cesarean delivery; or low umbilical artery pH (UA pH), or admission to neonatal unit (NU).

**Results:**

The indication for instrumental or cesarean delivery in women with type 1 and type 2 diabetes mellitus was more frequently based on fetal distress as compared to controls (adjusted OR 2.76 CI 1.74–4.40 and adjusted OR 2.31 CI 1.19–4.51, respectively). In comparison with the control group, infants of women with type 1 diabetes had an increased risk of UA pH < 7.20 (adjusted OR 1.88 CI 1.23–2.87) or UA pH < 7.10 (adjusted OR 3.35 CI 1.79–6.27). Also, infants of women with type 1 diabetes were at increased risk for admission to NU as compared to infants of control women (OR 8.07 CI 4.75–13.70).

**Conclusions:**

Women with type 1 and type 2 diabetes are at increased risk of fetal distress during labor as compared to controls.

## Background

Diabetes mellitus is a common medical disorder characterized by an absolute or relative deficiency of insulin, thereby causing a chronic state of hyperglycemia. It is associated with an increased risk of atherosclerosis and an increased prevalence of cardiovascular diseases [1]. Type 1 diabetes mellitus is caused by a destruction of the β-cells of the islands of Langerhans in the pancreas, which produce insulin. Type 2 diabetes mellitus is characterized by insulin resistance and impairment in insulin secretion. Gestational diabetes mellitus is defined as carbohydrate intolerance with onset or first recognition during pregnancy [1].

It is estimated that 2–8% of pregnancies involve women with diabetes of which approximately 87.5% are gestational diabetes, of the remainder 7.5% are type 1 diabetes and 5% are type 2 diabetes [2–5]. In addition, there is a rising prevalence of pregnancies complicated by diabetes mellitus. This is due to a reduction in the maternal age at onset of diabetes and also that women are tending to delay reproduction [6]. Other factors that may contribute are an increasing prevalence of obesity, increased intake of polyunsaturated fat and decreased physical activity [7–9].

Pregnancies complicated by diabetes mellitus have an increased risk of maternal mortality and morbidity [10]. Furthermore, there is an increased risk of adverse perinatal outcomes, including births defects, perinatal mortality and morbidity [10, 11]. It is suggested that higher rates of cesarean section (CS) result from failure to progress of labor, due to fetal macrosomia [12]. Data suggest that stillbirth and perinatal mortality may be increased as much as 3 times in women with pregestational diabetes compared to the general population [13]. In women with gestational diabetes these results varies widely [13–15]. It is hypothesized that fetal hypoxia, fetal acidemia and alterations in maternal and fetal metabolism may contribute to poor fetal outcome [16]. Therefore, the objective of this study is to evaluate the rate of intrapartum fetal distress in women with diabetes.

## Methods

### Study population

This retrospective case-cohort study was conducted at the VU University Medical Center, a tertiary care hospital in Amsterdam (The Netherlands) between March 2004 and February 2014. At the outpatient department, pregnant women with diabetes mellitus are monitored in accordance to a standardized evidence-based protocol by a multidisciplinary team consisting of a gynecologist, endocrinologist, diabetic nurse and a dietician [17].

Diabetes mellitus was classified in 3 different categories: type 1 diabetes, type 2 diabetes and gestational diabetes. The diagnosis of type 1 and type 2 diabetes was made prior to pregnancy in accordance to the Dutch guideline of internal medicine [18]. Pregnant women with one or more risk factors for gestational diabetes were screened and identified in accordance to the Dutch guideline of diabetes in pregnancy [17]. Risk factors for gestational diabetes included a history of gestational diabetes, macrosomia or unexplained intrauterine fetal death, BMI > 30 kg/m^2^, family history of type 2 diabetes (first-degree relatives), ethnic origin (South-Asian, African-Caribbean, Moroccan, Egyptian and Middle Eastern), polyhydramnios, estimated fetal weight > p95 and polycystic ovary syndrome. Screening for gestational diabetes was done by a 75-g oral glucose tolerance test. The diagnosis of gestational diabetes was based on a fasting plasma glucose level of ≥6.1 mmol/L or a 2-h level ≥ 7.8 mmol/L [19].

During pregnancy, type 1 and type 2 diabetes were treated with insulin. Insulin Aspart (Novorapid®) and Insulin Isofane (Insulatard®) were most commonly prescribed. Gestational diabetes was initially treated with diet and lifestyle recommendations. If this resulted in insufficient glycemic control, insulin was prescribed.

Measurements of blood glucose levels were performed on clinical assessment. Measurements of HbA_1c_ levels were done every four to six weeks [17]. During follow-up visits, glycemic control was evaluated and insulin was started or adjusted if necessary. Highest HbA_1c_ levels in the third trimester were used for the purpose of this study and categorized in < 42 mmol/mol (< 6%), 42–53 mmol/mol (6–7%) and > 53 mmol/mol (> 7%).

The study population included 479 pregnant women with diabetes mellitus and their infants. These women were matched to a control group that comprised 15,260 low to high risk women without diabetes who delivered at the VU University Medical Center over the same period of time. From 14 multiple-gestation pregnancies in women with diabetes mellitus and 647 multiple-gestation pregnancies in the control group, only first born infants were included in this study. This decision was based on the fact that the mode of delivery for the first born infant may directly influence the mode of delivery for the second born infant. In this respect, a randomized controlled trial by Barret et al. reported that in women planned for vaginal delivery for twin pregnancy already 7% underwent a cesarean section after their first born was delivered vaginally [20].

### Data collection

Maternal characteristics (maternal age, hypertensive disorders, BMI, lifestyle), pregnancy characteristics (gravidity, nulliparity, gestational age at delivery, obstetric history of CS), mode of delivery, indication for instrumental delivery or CS and neonatal outcomes (Apgar-scores, umbilical artery (UA) pH and neonatal unit (NU) admission) were obtained from patient records and compared with those from the control group. Pre-existent hypertension, pregnancy-induced hypertension and pre-eclampsia were defined as hypertensive disorder. Birthweight above p95 or above 4500 g was defined as macrosomia [17]. Neonatal unit admission included all infants residing in the Neonatal Intensive Care Unit, High Care Unit or Neonatology ward.

Data regarding the control group was obtained from the Landelijke Verloskunde Registratie (LVR). The LVR is a national population-based surveillance system covering all births of > 16 weeks gestation. It includes information on maternal characteristics, pregnancy, labor, delivery and neonatal outcomes.

The number and type of delivery of all women categorized as having diabetes were documented and compared with the control group. The indications for instrumental or cesarean delivery was analysed. Fetal distress was diagnosed by the clinician (gynecologist, resident, midwife) and was based on the fetus having a rapidly deteriorating or abnormal cardiotocographic (CTG) pattern and/or a fetal scalp pH < 7.20. After delivery, UA pH was determined to verify whether the clinical diagnosis of fetal distress was accurate. In accordance to the NICE guidelines, cut-off point of UA pH < 7.20 was defined as fetal distress [21]. Additional analysis was done using the cut-off point of UA pH < 7.10 due to its association with adverse neurological outcome [22].

### Statistical methods

Linear and logistic regression analyses were used to compare maternal and pregnancy characteristics as well as neonatal outcome. Risk of fetal distress and perinatal asphyxia from type 1, type 2 and gestational diabetes was determined by multiple regression analysis, adjusted for potential confounding factors such as age, smoking, parity, previous CS, hypertensive disorder, pre-eclampsia, prematurity, induction of labor and macrosomia. In additional analysis, the relation between HbA_1c_ levels and UA pH was examined. Results are described in terms of odds ratios (OR), with 95% confidence intervals (CI). All tests were two-sided and *p* values < 0.05 were considered statistically significant. Statistical analyses were performed with SPSS® version 21 (IBM, Chicago, IL, USA).

## Results

At the VU University Medical Center, 117 women with type 1 diabetes, 59 women with type 2 diabetes and 303 women with gestational diabetes were registered between March 2004 and February 2014.

Maternal and pregnancy characteristics are presented in Table 1. Our primary outcomes are summarized in in Tables 2, 3 and 4. Women with type 1 and type 2 diabetes were more likely to deliver by unplanned CS (type 1 diabetes *n* = 42, 35.9%, OR 3.92 CI; 2.55–6.03; type 2 diabetes *n* = 17, 28.8%, OR 3.03 CI; 1.61–5.72) than the control group (*n* = 2313, 15.2%). In all types of diabetes, 84 women underwent an instrumental delivery due to suspected fetal distress (Table 2). Fetal scalp blood samples were obtained in 21 women, 6 of which showed a pH below 7.20. In these cases an instrumental delivery was performed due to low pH. In all other cases (pH ≥ 7.20), the final decision was based on the interpretation of the CTG.

**Table 1:** Frequencies of maternal and pregnancy characteristics of women with diabetes mellitus and the control population

Maternal and pregnancy characteristics	Type 1 diabetes *(n = 117)*	Type 2 diabetes *(n = 59)*	Gestational diabetes *(n = 303)*	Control group *(n = 15,260)*
Age [y, mean ± SD]	32.9 ± 4.00	35.9 ± 4.86 **	35.5 ± 4.98 **	33.1 ± 4.95 *(n = 15,257)*
Primigravidity [n (%)]	50 (42.7)	12 (20.3) *	80 (26.4) **	6173 (40.5)
Nulliparity [n (%)]	61 (52.1)	17 (28.8) **	124 (40.1) ** *(n = 302)*	8131(53.4) *(n = 15,216)*
Previous CS [n (%)]	23 (19.7) *	22 (37.3) **	47 (15.5)	1808 (11.8)
Smoker [n (%)]	12 (10.5) * *(n = 114)*	2 (3.5) *(n = 57)*	19 (6.8) *(n = 280)*	592 (5.0) *(n = 11,873)*
BMI [kg/m^2^, mean ± SD]	25.1 ± 3.37 *(n = 105)*	31.6 ± 5.48 *(n = 53)*	28.1 ± 6.52 *(n = 275)*	insufficient data
HbA_1c_, mmol/mol (%) [mean ± SD]				
Trimester 1	50 ± 9.0 (6.76 ± 0.82) *(n = 95)*	51 ± 12.6 (6.81 ± 1.15) *(n = 38)*	38 ± 5.9 *** (5.67 ± 0.54) *(n = 24)*	not available
Trimester 2	40 ± 7.3 (6.35 ± 0.67) *(n = 112)*	47 ± 11.1 (6.42 ± 1.02) *(n = 54)*	39 ± 8.4 *** (5.72 ± 0.77) *(n = 96)*	not available
Trimester 3	48 ± 9.5 (6.54 ± 0.87) *(n = 111)*	47 ± 9.1 (6.42 ± 0.83) *(n = 53)*	40 ± 6.3 *** (5.81 ± 0.58) *(n = 268)*	not available
Hypertensive disorder [n (%)]	47 (40.9) ** *(n = 115)*	30 (51.7) ** *(n = 58)*	83 (28.9) ** *(n = 287)*	1378 (9.0)
Pre-eclampsia [n (%)]	13 (11.3) ** *(n = 115)*	6 (10.3) ** *(n = 58)*	26 (9.1) ** *(n = 287)*	367 (2.4)

Data are presented as mean ± standard deviation or number (%)

*= *p* < 0.01 as compared to control group

**= *p* < 0.001 as compared to control group

***= *p* < 0.001 as compared to type 1 diabetes

**Table 2:** Frequencies of obstetric interventions and outcomes in women with diabetes mellitus and the control population

Obstetric interventions and outcomes	Type 1 diabetes *(n = 117)*	Type 2 diabetes *(n = 59)*	Gestational diabetes *(n = 303)*	Control *(n = 15,260)*
Induction [n (%)]	53 (45.3) ‡	25 (42.4) †	132 (43.6) ‡	3515 (23.0) *(n = 15,255)*
Preterm birth [n (%)]	31 (26.5) *	19 (32.2) †	37 (12.2) †	2830 (18.6) *(n = 15,248)*
Delivery mode [n (%)]				
Spontaneous §	42 (35.9) §	22 (37.3) §	155 (51.2) §	9072 (59.4)
VE/FE	9 (7.7)	1 (1.7)	32 (10.6)	1796 (11.8)
Planned CS	23 (19.7) ‡	19 (32.2) ‡	55 (18.2) †	1902 (12.5)
Unplanned CS	42 (35.9) ‡	17 (28.8) †	58 (19.1) *	2313 (15.2)
Others	1 (0.9)	–	3 (1.0)	177 (1.2)
Clinical indication for instrumental or cesarean delivery[n (%)]				
Failure to progress	15 (12.9)	3 (5.2)	41 (13.8)	1991 (13.2)
Fetal distress	32 (27.6) ‡ *(n = 116)*	14 (24.1) † *(n = 58)*	38 (12.8) *(n = 298)*	1655 (11.0) *(n = 15,078)*

Data are presented as mean ± standard deviation or number (%)

VE, vacuum extraction; FE, forceps extraction; Others, breach delivery or fundus expression

*= *p* < 0.05 as compared to control group

†= *p* < 0.01 as compared to control group

‡= *p* < 0.001 as compared to control group

§= referent variable of logistic regression analysis

**Table 3:** Frequencies of neonatal outcomes in women with diabetes mellitus and the control population

Neonatal outcomes	Type 1 diabetes *(n = 117)*	Type 2 diabetes *(n = 59)*	Gestational diabetes *(n = 303)*	Control *(n = 15,260)*
Birthweight [g, mean ± SD]	3487.5 ± 755.6 †	3345.5 ± 936.4	3443.2 ± 702.1 †	3102.1 ± 975.6 *(n = 15,243)*
Macrosomia [n (%)]	45 (38.5) ‡	19 (32.8) ‡ *(n = 58)*	60 (19.8) ‡	695 (4.8) *(n = 14,518)*
UA pH [mean ± SD]	7.22 ± 0.09 † *(n = 104)*	7.23 ± 0.08 *(n = 51)*	7.24 ± 0.08 *(n = 247)*	7.24 ± 0.08 *(n = 8942)*
UA pH < 7.20 [n (%)]	38 (36.5) † *(n = 104)*	11 (21.6) *(n = 51)*	56 (22.7) *(n = 247)*	2186 (24.4) *(n = 8942)*
UA pH < 7.10 [n (%)]	13 (12.5) ‡ *(n = 104)*	2 (3.9) *(n = 51)*	12 (4.9) *(n = 247)*	372 (4.2) (n = 8942)
Apgar (after 5 min) < 7 [n (%)]	9 (7.8) *(n = 116)*	6 (10.3) *(n = 58)*	13 (4.4) * *(n = 298)*	1263 (8.3) *(n = 15,246)*
Admission to NU [n (%)]	71 (61.2) ‡ *(n = 116)*	27 (45.8) ‡ *(n = 59)*	83(28.0) ‡ *(n = 296)*	2813 (18.4) *(n = 15,257)*

Data are presented as mean ± standard deviation or number (%)

*= *p* < 0.05 as compared to control group

†= p < 0.01 as compared to control group

‡= p < 0.001 as compared to control group

**Table 4:** OR of primary outcomes of women with diabetes as compared to the control population

	Type 1 diabetes		Type 2 diabetes		Gestational diabetes	
	Crude OR (95% CI)	Adjusted OR* (95% CI)	Crude OR (95% CI)	Adjusted OR* (95% CI)	Crude OR (95% CI)	Adjusted OR* (95% CI)
Clinical indication for instrumental delivery or CS						
- Failure to progress	1.25 (0.71, 2.19)	1.03 (0.56, 1.90)	0.42 (0.13, 1.36)	0.46 (0.13, 1.59)	1.08 (0.77, 1.51)	0.95 (0.64, 1.39)
- Fetal distress	3.20 (2.10, 4.89)	2.76 (1.74, 4.40)	2.36 (1.28, 4.34)	2.31 (1.19, 4.51)	1.20 (0.85, 1.70)	1.08 (0.74, 1.59)
UA pH < 7.20	1.78 (1.19, 2.66)	1.88 (1.23, 2.87)	0.85 (0.44, 1.66)	1.03 (0.52, 2.04)	0.91 (0.67, 1.23)	0.93 (0.68, 1.29)
UA pH < 7.10	3.29 (1.82, 5.94)	3.35 (1.79, 6.27)	0.94 (0.23, 3.88)	1.02 (0.24, 4.32	1.18 (0.65, 2.12)	1.26 (0.67, 2.37)
Admission to NU	6.98 (4.79, 10.16)	6.41 (3.84, 10.68)	3.73 (2.23, 6.24)	2.36 (1.09, 5.10)	1.72 (1.33, 2.23)	1.90 (1.20, 3.03)

*Adjusted for maternal age, smoking, nulliparity, previous CS, hypertensive disorder, pre-eclampsia, prematurity, induction and macrosomia

Mean UA pH was slightly lower in women with type 1 diabetes (7.22 ± 0.09, OR -0.28 CI; − 0.04- -0.01) compared to the control group (7.24 ± 0.01). The risk of UA pH < 7.20 or UA pH < 7.10 in type 1 diabetes compared with the control group was significantly increased (Table 4). UA pH < 7.20 and UA pH < 7.10 rates in type 1 diabetes were also increased compared to gestational diabetes, (adjusted OR 2.01 CI; 1.20–3.38 and adjusted OR 2.64 CI; 1.13–6.21, respectively).

In addition, when fetal distress was the indication to instrumental delivery, higher rates of UA-pH < 7.20 (*n* = 17; 56.7%) or UA-pH < 7.10 (*n* = 6; 20%) were found in women with type 1 diabetes as compared to the control group (*n* = 531, 43.9% adjusted OR 2.81 CI 1.10–7.20 and *n* = 124, 10.3% adjusted OR 4.47 CI 1.32–15.19 respectively).

In additional analysis, a significant relationship between maternal HbA_1c_ levels in the third trimester of pregnancy and neonatal UA pH was observed. Infants of women with HbA_1c_ levels > 53 mmol/mol were at increased risk of UA pH < 7.20 (*n* = 17, 40.5%, adjusted OR 2.49 CI; 1.12–5.54) compared to women with HbA_1c_ levels < 42 mmol/mol (*n* = 40, 21.2%). When comparing UA pH < 7.10 in women with HbA_1c_ levels > 53 mmol/mol and < 42 mmol/mol this risk was also increased (*n* = 6, 14.3% and *n* = 9, 4.8% respectively, adjusted OR 3.94 CI; 1.15–13.54). Rates of UA pH < 7.20 or < 7.10 in women with HbA_1c_ levels within the range 42–53 mmol/mol and HbA_1c_ levels < 42 mmol/mol did not differ significantly.

Higher rates of NU admission were found in infants of women with type 1 and type 2 diabetes than those in the control group (Table 3). Since the relation between NU admission and prematurity is well-known, subsequent analyses were performed by excluding infants who were born before 37 weeks of gestation. It appeared, the risk for NU admission was more explicit in type 1 and type 2 diabetes (type 1 diabetes *n* = 43, 50% NU admission, OR 8.07 CI; 4.75–13.70; type 2 diabetes *n* = 14, 35% NU admission, OR 5.02 CI; 2.31–10.91). Furthermore, the risk of admission to NU increased significantly, when UA pH decreased (UA pH < 7.20, OR 1.32 CI; 1.13–1.55; UA pH, < 7.10 OR 2.63 CI; 1.99–3.46; UA pH < 7.00, OR 9.07 CI; 4.85–16.95).

## Discussion

### Main findings

In our study, the prevalence of fetal distress as clinical indication for instrumental or cesarean delivery was higher in women with type 1 (*n* = 32, 27.6%) and type 2 diabetes (n = 14, 24.1%) compared to the control group (*n* = 1655, 11.0%, *p* < 0.01). Furthermore, UA pH analysis showed a high prevalence of UA pH < 7.20 (*n* = 38, 36.5%) in infants of mothers with type 1 diabetes compared to the control group (*n* = 2186, 24.%, p < 0.01). In addition, rates of infants with UA pH < 7.10 in women with type 1 diabetes (*n* = 13, 12.5%) and the control group (*n* = 372, 4.2%) were significantly higher (p < 0.01). All results were confirmed by multiple regression analysis, adjusted for confounding factors as age, parity, previous CS, smoking, hypertensive disorder, pre-eclampsia, prematurity, induction of labor and macrosomia. These results suggest that infants of women with type 1 diabetes are at increased risk to suffer from fetal distress during delivery compared to the control group.

A relationship was found between high HbA_1c_ levels and low UA pH. Infants of women with HbA_1c_ levels > 53 mmol/mol were over 2.5 times more likely for UA pH < 7.20 or UA pH < 7.10 than women with HbA_1c_ levels < 42 mmol/mol, suggesting that poorly regulated diabetes results in higher rates of neonatal asphyxia. Furthermore, lower umbilical artery pH were significantly associated with higher rates of NU admission. Not unexpectedly, there was a higher prevalence of hypertensive disorders in all diabetic women. Hypertension in pregnancy is a well-known risk factor for perinatal asphyxia [23]. Our results remained unchanged after adjustment for hypertensive disorders.

### Strengths and limitations

Our retrospective case-cohort study covers a period of 10 years, thereby comprising a large population of women with diabetes. Our study is the first that has systematically evaluated perinatal outcomes of women with type 1, type 2 and gestational diabetes mellitus. The results from comparative analysis were confirmed by multiple regression analysis. In addition, our study was done in a tertiary care hospital. Thereby, the control group consisted of women with low to high risk of perinatal complications. Differences in rates of fetal distress or UA pH in women with diabetes mellitus in comparison with low-risk controls may be more explicit.

Our study had some limitations. Hospital-based administrative databases are subject to errors of omission and coding errors. A potential for under-ascertainment and misclassification bias exist. We tried to minimized this risk by double checking the patients charts. The diagnosis of fetal distress was based on the clinical assessment of the gynecologist or midwife. We lacked objective data on CTG analyses, and only in a small number of cases was the decision for instrumental delivery based on low fetal scalp pH (< 7.20). HbA1_c_ levels were used as unit of measure for glucose regulation. It is ambiguous whether HbA1_c_ levels are a result from continuously variable or constant glucose levels during pregnancy. Also, we lacked data on glucose regulation prior to delivery. A cohort study performed in a single hospital may limit the capacity to draw conclusions from results, since risk for gestational diabetes or fetal distress may differ between populations and obstetrical management of diabetes during pregnancy may differ between hospitals.

### Interpretation

There are other studies that describe the relation between fetal hypoxia and diabetes mellitus. A large population-based study from Lower Saxony in Germany recently reported high rates of UA pH < 7.00 in women with pregestational diabetes compared to controls (OR 2.481). The rate of UA pH 7.00–7.20 was also increased (OR 1.59). However, no distinction was made between type 1 and type 2 diabetes, and no multiple regression analysis was performed [24]. Moreover, in a prospective cohort study by Olofsson et al. [25]. a slightly increased risk for pH < 7.26, obtained by fetal scalp blood testing, and UA acidosis was reported in diabetic (*n* = 46) compared to non-diabetic pregnancies (n = 46). Also, Reif et al. [26]. confirmed lower UA pH in women with diabetes (*n* = 57) in comparison with the control group (*n* = 114) (7.215 vs. 7.250*, p* = 0.007). Nevertheless, in both studies, the sample size was too small to distinguish between type 1, type 2 and gestational diabetes. A prospective study comparing UA pH and nucleated red blood cells in women with diabetes (*n* = 68) and healthy controls (*n* = 410), also showed low UA pH (7.22 vs. 7.24, *p* = 0.004). However, delivery mode differed within both groups and they lacked multiple regression analysis [27]. Klemetti et al. [28] examined the trends in glycaemic control among type 1 diabetic patients and their relation to adverse perinatal outcome. Main result were an increasing trend of HbA_1_C levels prior to delivery and their negative correlation with UA pH. This is in accordance with our results and emphasizes the importance of good glucose regulation .

There are other findings in diabetic pregnancy that support our results. In a study by Teramo et al. [29], it was found that fetal erythropoietin (EPO) concentrations in macrosomic infants of diabetic mothers were elevated. Since tissue hypoxia is the major stimulus of EPO, this also implies a relationship between diabetes and fetal distress. Likewise, EPO levels in the umbilical cord blood are higher after uncomplicated vaginal birth than after elective CS [30]. This is explained by reduced placental perfusion caused by prolonged uterine contractions in combination with maternal bearing down efforts [31]. Since diabetes mellitus is associated with an increased prevalence of vascular diseases, high rates of fetal distress may possibly be explained by vascular changes in the placental circulation, causing reduced placental perfusion. This hypothesis was emphasized by Jones et al., demonstrating reduced placental fractional power Doppler signal in diabetic compared to healthy pregnancies [32].

Several studies have reported abnormal development and functioning of the fetoplacental vascular system, such as increased vascular permeability and increased angiogenesis. [33, 34] Diabetic placentas are often hypertrophied, with possible alterations in branching patterns, villous surface area an villous thickness. [34–36] Calderon et al. [36] reported that the higher the glycemic level, the lower the number of terminal villi, suggesting placenta inadequacy to ensure maternal-fetal exchanges and fetal oxygen delivery.

In cases of acute hypoxia, calcifications, fibrin deposition and fibrosis, due to membrane laminar necrosis and villous infarction, are often observed. [37] A small study of macroscopic placental changes in diabetes mellitus has shown that all women demonstrated some of those changes on the maternal or fetal surface of the placenta. [38] Those findings have their limitations, because they may occur in uncomplicated pregnancies as well. [37] However, this may also indicate that fetuses of women with diabetes may suffer from hypoxia prior to delivery.

## Conclusion

Type 1 and type 2 diabetes are associated with an increased risk of fetal distress during delivery as clinical indication for instrumental or cesarean delivery. Moreover, type 1 diabetes is significantly associated with lower UA pH and higher rates of NU admission. Future studies should examine placental abnormalities in relation to UA pH and NU admission. Also, it should be examined whether a strict glucose regulation during pregnancy reduces the prevalence of placental vascular abnormalities and vasculopathy, and thereby reduces the rates of peripartum hypoxia. Consequently, enhanced information about the relation between glucose regulation, obstetrical management and neonatal outcome can be given to diabetic women prior to labour and delivery.

## Abbreviations

BMI: body mass index
CI: confidence interval
CS: cesarean section
CTG: cardiotocography
EPO: Erythropoietin
NU: neonatal unit
OR: odd ratio
UA: umbilical artery
